# Key Events Participating in the Pathogenesis of Alcoholic Liver Disease

**DOI:** 10.3390/biom7010009

**Published:** 2017-01-27

**Authors:** Fernando Magdaleno, Chuck C. Blajszczak, Natalia Nieto

**Affiliations:** Department of Pathology, University of Illinois at Chicago, 840 S. Wood St., Suite 130 CSN, MC 847, Chicago, IL 60612, USA; fmverduz@uic.edu (F.M.); cblajs2@uic.edu (C.C.B.)

**Keywords:** damage-associated molecular patterns, inflammasome, iron, lipid peroxidation, macrophages, neutrophils, pathogen-associated molecular patterns

## Abstract

Alcoholic liver disease (ALD) is a leading cause of morbidity and mortality worldwide. It ranges from fatty liver to steatohepatitis, fibrosis, cirrhosis and hepatocellular carcinoma. The most prevalent forms of ALD are alcoholic fatty liver, alcoholic hepatitis (AH) and alcoholic cirrhosis, which frequently progress as people continue drinking. ALD refers to a number of symptoms/deficits that contribute to liver injury. These include steatosis, inflammation, fibrosis and cirrhosis, which, when taken together, sequentially or simultaneously lead to significant disease progression. The pathogenesis of ALD, influenced by host and environmental factors, is currently only partially understood. To date, lipopolysaccharide (LPS) translocation from the gut to the portal blood, aging, gender, increased infiltration and activation of neutrophils and bone marrow-derived macrophages along with alcohol plus iron metabolism, with its associated increase in reactive oxygen species (ROS), are all key events contributing to the pathogenesis of ALD. This review aims to introduce the reader to the concept of alcohol-mediated liver damage and the mechanisms driving injury.

## 1. Introduction

Alcoholic liver disease (ALD) is a leading cause of morbidity and mortality worldwide. It ranges from fatty liver to steatohepatitis, progressive fibrosis, cirrhosis and hepatocellular carcinoma. ALD refers to a number of symptoms/deficits that contribute to the ever-degrading state of the liver. These include characteristic features such as steatosis, inflammation, fibrosis and cirrhosis, which, when taken together, either sequentially or simultaneously account for the progression of liver disease [1]. The most prevalent forms of ALD are alcoholic fatty liver, acute and chronic alcoholic hepatitis (AH) and alcoholic cirrhosis. Disease progression occurs as patients continue to drink. Ethanol can interact with other risk factors to contribute to other forms of liver disease such as viral Hepatitis B or C [2] and non-alcoholic steatohepatitis, substantially increasing the risk to develop liver cirrhosis. Three candidate genes, *patatin-like phospholipase domain-containing protein 3*, *transmembrane 6 superfamily member 2* and *membrane-bound O-acyltransferase domain-containing 7*, have been identified to play a role in the susceptibility of developing alcohol-related liver injury [3,4]. However, the complex pathogenesis of ALD influenced by host and environmental factors is currently only partially understood. To date, lipopolysaccharide (LPS) translocation from the gut into the portal blood, aging, gender differences, increased infiltration of neutrophils and bone marrow-derived or peritoneal macrophages, along with alcohol plus iron metabolism, both of which increase reactive oxygen species (ROS) production, are considered key events contributing to the pathogenesis of ALD.

Importantly, ethanol is the substance of abuse most used worldwide; as such, the prevalence of ALD is widespread. Regionally, there are differences in the causes and onset of the disease; however, treatment universally begins the same way, with abstinence. This review aims to introduce the reader to hepatic alcohol-related damage, with the mechanisms of injury-response and the crucial players driving inflammatory infiltration and/or cell activation that exacerbate hepatic tissue deterioration.

## 2. Alcoholic Fatty Liver

The liver is a highly metabolic organ [5]. It acts as a pivot point between the fasting and feeding states and, as such, has both catabolic and anabolic capabilities. These processes are implicated when an excess of alcohol results in inadequate metabolism, endoplasmic reticulum stress and increased fatty acid and triglyceride synthesis, all of which lead to steatosis. The peroxisome proliferator-activated receptor α is critical for alcohol metabolism and when down-regulated or depleted, results in massive fat accumulation [6,7]. Sterol regulatory element-binding proteins (SREBP) and 5′ adenosine monophosphate-activated protein kinase (AMPK) are also implicated in ALD since they play an important role in regulating lipid metabolism and their expression is altered under alcohol consumption [8,9]. Down-regulation of AMPK by alcohol decreases its ability to inactivate SREBP and increases adenylyl cyclase activity, contributing to steatosis [10]. Recent advances in the field have shown that dietary lipids such as saturated and unsaturated fat play a critical role in alcohol-induced liver pathology. For a careful summary of recent advances on the role of dietary lipids in ALD, the reader is referred to recent work from Kirpich et al. [11].

Lipid accumulation is a critical feature in ALD; it also represents the early stage of the disease and it may be accompanied by inflammation, fibrosis, cirrhosis and hepatocellular carcinoma. Importantly, steatosis will develop in the majority of individuals who regularly consume alcohol in excess of 40 g/day [3]. In 10%–35% of drinkers, the presence of steatosis may be complicated by the development of inflammation and fibrosis where cirrhosis will likely develop in about 10%–15% of these patients [12] and 1%–2% of them progress to hepatocellular carcinoma (HCC) [13].

## 3. Alcoholic Hepatitis

Progressive inflammatory liver injury due to ethanol intake leads to AH, characterized by poor liver function, ductular reaction [14,15], high levels of LPS and impaired hepatocyte proliferation [16]. All these events, along with sterile and non-sterile inflammation [17], are mechanisms involved in the pathogenesis of the disease. AH has a significant short-term mortality. Key features are a significant increase in proinflammatory cytokines like tumor necrosis factor α (TNFα) and interleukins (IL)-1 and -6. Alcohol abstinence has demonstrated to benefit these patients by improving the prognosis in early and advanced stages [18]. Pentoxifylline and prednisolone improve short-time survival of AH and continue to be the main treatment for these patients, although corticosteroids could be detrimental since they increase the risk of bleeding and infections [19]. Thus, assessment of early response to corticosteroids, identification of novel biomarkers and/or unique drugs targets for the treatment of moderate and severe AH are still required to adequately monitor and treat this disease.

## 4. Alcoholic Cirrhosis

Excess alcohol consumption is the most frequent cause of cirrhosis in Europe [20]. In the United States, the prevalence of cirrhosis is 0.27%, corresponding to 633,323 adults in 2015 [21], higher than previously estimated. Thus, liver cirrhosis is an important public health concern and a significant cause of morbidity and mortality worldwide. In most cases, in addition to alcohol consumption, lifestyle, gender, ethnic and socioeconomic factors may contribute or synergize in the progression of the disease.

In patients with decompensated ALD, cirrhosis is more frequent and features of hepatocellular damage, lobular inflammation, hepatocellular ballooning degeneration and hepatocyte drop-out, megamitochondria and the appearance of Mallory–Denk bodies are more dominant [18]. Liver cirrhosis leads to severe complications, such as variceal bleeding, ascites and hepatic encephalopathy. When cirrhosis occurs, the sinusoidal space ensuring blood supply to the parenchyma decreases, sinusoidal endothelial cell fenestrations collapse and numerous new vessels formed around the cirrhotic nodules bypass the obstructed normal route. This vascular proliferation contributes to the remodeling of the liver architecture, collateral flow and portal hypertension. The latter is a frequent and severe complication of cirrhosis, a leading cause of death and an indicator for liver transplantation [22].

## 5. Key Players in ALD

### 5.1. ROS and Lipid Peroxidation

Oxidant stress plays a dominant role in the pathogenesis of ALD, as multiple studies have shown that generation of ROS is key for the progression of fatty liver to steatohepatitis and cirrhosis [23,24,25,26,27]. They induce pathological changes such as steatosis, inflammatory cell infiltration, hepatomegaly, fibrosis and cirrhosis [28]. Over time, these cellular events increase and, again, the only well-established treatment is abstinence.

There are three main alcohol metabolic pathways in the liver: alcohol dehydrogenase (ADH) [29], microsomal ethanol oxidation system (cytochrome P450 2E1) [30] and catalase [31]. Under conditions of chronic alcohol intake, the microsomal cytochrome P450 becomes activated and produces large amounts of aldehyde, 1-hydroxyethyl radical and other free radicals [32]. Once generated, free radicals interact with polyunsaturated fatty acids found in membranes throughout the cell and its components, generating lipid peroxidation end-products and protein adducts. Over time, they lead to necrosis and, ultimately, significant liver damage [33].

A healthy liver can metabolize alcohol through a series of steps that result in an increase in fat and acetyl-CoA, which, in turn, up-regulate mitochondrial metabolism to compensate for the biochemical changes. The intermediary steps require the consumption of oxidized nicotinamide adenine dinucleotide (NAD)^+^ and reduced nicotinamide adenine dinucleotide (NADH) to allow ADH to break down alcohol into acetaldehyde and subsequently undergo oxidation via aldehyde dehydrogenase (ALDH2) to convert acetaldehyde to acetic acid. This process varies depending on gender—low ADH activity in women—and alters the ratio of NAD^+^/NADH leading to accumulation of acetyl-CoA and fat [28]. The oxidative metabolism of alcohol alters the NAD^+^/NADH ratio by increasing the amount of NADH, a phenomenon typically called “swift increase in alcohol metabolism” [34]. As a result, fatty acid oxidation is blocked and the liver experiences an increase in lipogenesis, causing the mitochondria to try to compensate [35]. Lieber and colleagues have meticulously described the precise mechanism whereby alcohol induces toxicity in the liver [36,37], reinforcing that continuous exposure of hepatocytes to ethanol activates complex signaling pathways that lead to liver injury and death. Thus, ethanol metabolism directly impairs hepatocyte function, leading to lipid accumulation and apoptosis, both of which are associated with ALD [38].

### 5.2. Sex Differences

Sex is one of many factors that influences alcohol oxidation [39]. The presence of acetaldehyde in the blood depends on the ability of ALDH2 to metabolize it, which is sex-dependent. Animal studies have shown marked sex differences in hepatic ADH activity [40], being up to 100% higher in females than males. This leads to an arterial acetaldehyde burst shortly after alcohol consumption and enhanced vulnerability of women to develop ALD. Despite the knowledge that women are at higher risk of ALD, numerous questions about sex differences in ethanol metabolism and the role of ADH isoforms on different populations are still open.

### 5.3. Aging

Aging is the deterioration found in all organisms and contributes to increased susceptibility to any disease. Individuals face a number of insults over the course of their lifespan and the unique combination of changes allows one disease over another to manifest. This holds true in the liver as indeed it aggravates ALD. Changes in the liver can be associated with cell death, ROS damage to DNA and proteins, as well as the appearance of mutations or instability and changes in the composition of the lipid bilayer and lipid rafts. A study looking at mitochondrial DNA (mDNA) damage, found that just 3% of non-alcoholic patients had significant changes in their mDNA; yet, 24% of alcoholic patients had mDNA deletion and 85% of patients with microvesicular steatosis had at least one deletion with many individuals having several [41]. Therefore, alcohol is believed to be primarily responsible for these differences and can be explained by the increase in oxidative stress building up and causing increasingly more mDNA mutations. Each of these mutations only affects a small number of mitochondria at a time; with persistent damage it can culminate in impaired mitochondrial efficiency and oxidative phosphorylation. The depletion of energy impacts the cell function and can initiate an inflammatory or oncogenic response. Mansouri et al. [41] show that this increased stress induces more mutations, specifically deletions and rearrangements of the mDNA. Patients that have these mDNA deletions are often found to have microvesicular steatosis that can be fatal [42]. Interestingly, they are largely not found in age-matched non-alcoholic patients.

The liver undergoes a number of changes as it ages, including hepatocytes presenting an unhealthy phenotype, lower proliferative potential, increase in fat and cholesterol, reduced low-density lipoprotein (LDL) metabolism and inflammation [43,44]. A protein recently of interest as a potential therapeutic target for the treatment of ALD is sirtuin-1 (SIRT1), a histone deacetylase known to be involved in lipid accumulation and metabolism. Ramirez et al. [43] have shown that in aged mice fed with an ethanol-plus ethanol binge(s) diet, SIRT1 decreased in both hepatocytes and hepatic stellate cells (HSC), contributing to significant liver injury and fibrosis. SIRT1 has been shown to play a protective role against ALD and non-alcoholic fatty liver disease (NAFLD) by changing the acetylation status of target molecules to seemingly inhibit inflammation and steatosis. Similar results have been observed in older patients who also experience less blood flow through the liver, decreased liver volume by 20–40% and tend to take medications contraindicated with alcohol consumption [45]. All these factors make the liver of these patients more susceptible to ALD.

Increased production of ROS in the presence of alcohol results in mitochondrial damage coexisting with depletion of the antioxidant defense, especially glutathione. In addition, aging increases gut leakiness due to loss of transepithelial electrical resistance [46,47]. Increased permeability induces bacterial and LPS translocation and activation of resident Kupffer cells (KC) to clear circulating LPS [44]. Although several events have been identified to be responsible for the increased susceptibility of aged livers to ALD, the exact underlying mechanisms are still unclear.

### 5.4. Iron

Iron is essential for life in all mammalian organisms as it is a co-factor of various proteins, enzymes and reactions. It has a key role in oxygen transport and in enzymes involved in mitochondrial respiration, DNA biosynthesis and the citric acid cycle through its ability to change its redox state [48]. Since iron has a limited capacity to be excreted, it can build up in the body with potential lethal consequences. Because iron is a very potent oxidant, excess iron is sufficient to worsen and accelerate the course of ALD since iron-induced oxidative damage of macromolecules by ROS (produced by the Fenton reaction [49]) leads to lipid peroxidation and to the release of toxic long-lived lipid peroxidation by-products [50], which bind and damage proteins and DNA by forming protein or DNA adducts [51]. Likewise, plasma membrane damage, increase in lysosome instability, decrease in cytochrome activity and mitochondrial damage are the main targets of iron toxicity.

Hepatic iron overload has been reported in patients with advanced ALD as it hits the liver in concomitance with alcohol and ROS. Iron favors synthesis of cytokines and other fibrogenic or hepatotoxic mediators. However, it is not definitively known whether it is the effect of alcohol that is responsible for such overload [52]. Considering that hepcidin expression in the liver inhibits iron absorption from the diet and the release of iron from its storage, it is feasible that modulation of hepcidin synthesis and activity or hepcidin hormone-replacing strategies may become therapeutic options for patients with ALD in the future [53].

### 5.5. Inflammation

#### 5.5.1. Neutrophils

Many insults can lead to liver injury. Due to its great replicative ability, the liver can restore to its normal state on its own. But with extended exposure to a stressor, the liver loses the ability to cope. After the initial insult resulting in injury, proinflammatory cells increase or extravasate into the liver and exacerbate inflammation.

These populations include resident macrophages or KC, circulating macrophages, neutrophils, natural killer (NK) and natural killer T (NKT) cells [6,54,55,56,57]. Many inflammatory cytokines, such as TNFα, IL-1β, monocyte chemoattractant protein-1 (MCP-1) and IL-6 are also produced in and outside the liver. Alcohol increases neutrophil recruitment to the liver, which is easily detectable by up-regulation of neutrophil markers Lymphocyte antigen 6 complex locus G6D (Ly6G), Myeloperoxidase (MPO) and E-selectin [54]. 

The contribution neutrophils make to ALD is in the production of harmful mediators they release after their recruitment such as H_2_O_2_, elastase, chloramine and proteinase-3. Increased expression of toll-like receptors (TLR) 4, 2 and 9 influence neutrophil dysfunction without any effect on phagocytic activity [58]. These receptors signal through a number of pathways including the myeloid differentiation primary response gene 88 (MyD88)-TRIF and interferon regulatory factor 3 (IRF3). All of them have been implicated in ALD as well as other liver diseases and seem to play distinct yet overlapping roles. In a study looking at mice null for *Tlr2* and *Tlr9*, a significant decrease in the number of neutrophil-recruiting chemokines (C-X-C motif) ligand 1, 2 and 5 (CXCL1, CXCL2 and CXCL5) was observed [59]. Moreover, IRF3 has been found to contribute to ALD, inducing apoptosis via the up-regulation of Bcl2 associated X, apoptosis regulator in hepatocytes [60], therefore linking endoplasmic reticulum stress signaling with hepatocyte apoptosis. Distinctly, IRF3 plays two separate roles in this situation with it being up-regulated in macrophages after stimulation by LPS, but also involved in the direct ethanol-induced apoptosis of hepatocytes [60]. Conversely, apoptosis can block neutrophil recruitment via a TLR2- and TLR9-mediated mechanism [61], demonstrating that hepatocyte apoptosis in response to alcohol is a hallmark in ALD.

Besides neutrophils, other proteins involved in deleterious inflammation referred to as “alarmins” can amplify alcohol-mediated injury such as high-mobility group box 1 (HMGB1) [62,63], Mincle [64], IL-1β, IL-1α, IL-33 [65] and S100 proteins [66]. These dual-function alarmins share conserved regulatory mechanisms, posttranslational modifications and enzymatic processing that govern their intracellular and extracellular function. They can either exert a beneficial cell housekeeping effect or provoke uncontrolled inflammation [67]. Thus, their importance in inflammation has to be highlighted in ALD [68,69].

#### 5.5.2. Macrophages

Proinflammatory macrophages include KC and infiltrating bone-marrow and peritoneal monocytes. They are associated with recruitment of additional pro-inflammatory cells (including neutrophils), activation of myofibroblasts and hepatocyte apoptosis, production of cytokines and generation of ROS, all of which contribute to liver damage and disease progression. On the other hand, anti-inflammatory or restorative macrophages help break down extracellular matrix and favor disease resolution [70]. The macrophage/monocyte plasticity has been mainly studied in in vitro models that do not accurately mimic the complexity of the in vivo environment [71].

In ALD, macrophages favor the inflammatory reaction and fibrosis. LPS translocates from the gut to the portal blood, enters the liver and activates TLRs and receptor for advanced glycation end-products (RAGE) receptors on KC and, to some extent, in HSC, causing an increase in fibrosis both indirectly by stimulating KC [72] to produce ROS, which in turn activate HSC [73], or directly by activating HSC. Signaling via phosphatidylinositide 3-kinase (PI3K)/Protein kinase B (pAkt) and alarmins such as HMGB1 triggers and increases intracellular and extracellular collagen-I [74]. Thus, the gut-to-liver interaction plays a significant role in the setting of ALD.

Several molecules within the intestinal epithelium block bacterial translocation, reducing hepatic inflammation and steatosis. Osteopontin (OPN), an extracellular matrix protein whose expression is associated with steatosis, inflammation and fibrosis, protects by maintaining the epithelial barrier function, providing mucosal defense, preventing sepsis and the inflammatory response [75]. As a proof of concept, administration of milk-derived osteopontin prevented alcohol-induced liver injury by maintaining gut integrity and averting hepatic inflammation and steatosis [76].

All of these events do not occur alone however. There is crosstalk between cells and organs allowing for the cumulative response and physiological manifestation observed in patients. The link between macrophages and neutrophils occurs early on in the response to injury [77]. Resident macrophages sense damage that has occurred to the liver and release chemokines, CXCL1 and macrophage inflammatory protein 2 (MIP-2) or (Chemokine (C-X-C motif) ligand 2, CXCL2) [1,78]. These are produced through the activation of the TLRs and can be produced through both the MyD88 and TRIF pathways, though CXCL1 can only be produced by the MyD88 pathway [79]. Li et al. [80,81,82] have proposed that MyD88 can activate the transforming growth factor β (TGFβ)-activated kinase 1 (TAK1) and mitogen-activated protein/extracellular signal–regulated kinase (ERK) kinase kinase 3/nuclear factor kappa B (NFκB) dependent signaling pathways, suggesting that TLR-dependent macrophage activation links alcohol-induced cell death to subsequent inflammatory responses in ALD.

#### 5.5.3. The Inflammasome

Inflammasomes are cytoplasmic multiprotein complexes that can sense danger signals from damaged cells and pathogens and assemble to mediate caspase-1 activation [83], which proteolytically activates IL-1β and IL-18 [84,85,86], as a result of tissue damage or cellular stress. The dogma is that the inflammasome requires two signals for full activation. The first is believed to be through TLR4 and TLR9, both sensitive to a number of direct ligands including LPS, DNA and HMGB1. The second signal appears to derive from injured cells (hepatocytes e.g., host DNA) that stimulate caspase-1 activation [87].

However, the role of the inflammasome in the development and progression of ALD deserves further exploration since inflammation is a central component not only in ALD, but also of most chronic liver diseases and contributes to progressive liver damage. Several members of the nucleotide-binding and oligomerization domain and leucine-rich-repeat-containing proteins (NLR) family exhibit inflammasome activity, including NLRP1, NLRP3, NLRP6 or NLRC4. The relevance of NLRP3 in liver disease has focused on the mechanisms limiting inflammasome activation and on pharmacological targeting of NLRP3 [88], as it is involved in ethanol-induced liver injury by driving liver inflammation and neutrophil infiltration [89].

Since IL-1β and IL-18 are pleiotropic cytokines produced by activated NLRP3 in macrophages that affect inflammatory and immune responses and have an important influence in the pathogenesis of many acute and chronic inflammatory diseases [90,91,92], including ALD, identifying the mechanisms driving activation of the inflammasome either directly in HSC, KC and hepatocytes, or indirectly through cell death and increased exposure to pathogen-associated molecular patterns (PAMPs), would be critical to advance the field of research on ALD.

After alcohol-exposure, KC-derived IL-1β production, likely triggered by NLRP3 activation, recruits and activates hepatic invariant NKT cells, subsequently promoting liver inflammation, neutrophil infiltration and ALD [89]. Indeed, the inflammasome is highly activated in the ascitic fluid of cirrhotic patients, which may explain the exacerbated inflammatory response observed in these individuals under non-infected conditions [93]. Thus, emerging evidence suggests that both KC and inflammasome activation contribute to the pathogenesis of ALD.

#### 5.5.4. PAMPs and Damage-Associated Molecular Patterns

Multiple receptors can sense PAMPs and Damage-Associated Molecular Patterns (DAMPs) to activate inflammatory cascades. PAMPs are mostly derived from the gut, due to alterations in gut microbiota composition and/or increased intestinal permeability [94]. DAMPs are mostly derived from damaged cells and include HMGB1, adenosine triphosphate (ATP), uric acid, cholesterol crystals, DNA fragments and fatty acids. The abundance of each DAMP is dependent on the type of injury and metabolic events [95]. For example, uric acid and ATP are required for activation of the inflammasome and IL-1β production for the development of inflammation in AH [96]. Moreover, exposure of hepatocytes and KCs to cholesterol crystals have been suggested in the NLRP3 activation during hepatic inflammation [97,98]. However, a subset of activators has emerged as effector molecules of liver damage such as HMGB1.

HMGB1 was discovered over 30 years ago, as an endogenous inflammatory mediator and now serves as a prototype for the class of inflammatory activators tagged as “alarmins” [99]. HMGB1 is a key mediator of inflammation, macrophage proliferation, TNFα production and collagen-I deposition. Among other receptors, HMGB1 binds to TLRs and RAGE, both of which are implicated in liver disease [100,101]. HMGB1 is secreted in response to tissue injury or local and systemic inflammatory responses [102] and drives the profibrogenic response to liver injury [103,104]. Thus, it has been proposed that, during fibrogenesis, hepatocytes and KC increase and secrete [105] HMGB1 that acts either as an autocrine signal targeting themselves, creating a feedback loop, or as a paracrine signal targeting hepatic stellate cells.

HMGB1 can be released either passively by dying cells or actively during an inflammatory response [99] and from LPS-activated macrophages during sepsis [106]. It is normally found in the nucleus but can undergo post-translational modifications and, as a result, it translocates to the cytosol and is ultimately secreted. HMGB1 has been directly linked to the pathogenesis of ALD. Looking at both human samples and mouse models, the Nieto Lab found that HMGB1 was increased in disease progression and ablation of *Hmgb1* in hepatocytes protected from ALD in mice [107], suggesting that HMGB1 could be a pivotal regulator and perhaps a biomarker of liver injury. Likewise, a novel HMGB1 neutralizing chimeric antibody has been shown to attenuate acetaminophen-induced liver injury and post-injury inflammation in mice [99], demonstrating that HMGB1-specific therapy reduces toxicity and inflammation in acute liver injury and in chronic liver diseases.

Not only HMGB1, but also a large number of molecules and receptors, are involved in this process and therefore could be potential therapeutic targets in ALD. The TLR family of receptors is known to be involved in the inflammatory response. Additionally, the *N*-methyl-d-aspartate (NMDA) receptor has recently been found to be involved after resident macrophages of the liver were found to express it and, when activated, it suppresses pro-caspase-1 and pro-IL1-β to block inflammation [108].

## 6. Conclusions

**Figure 1:**
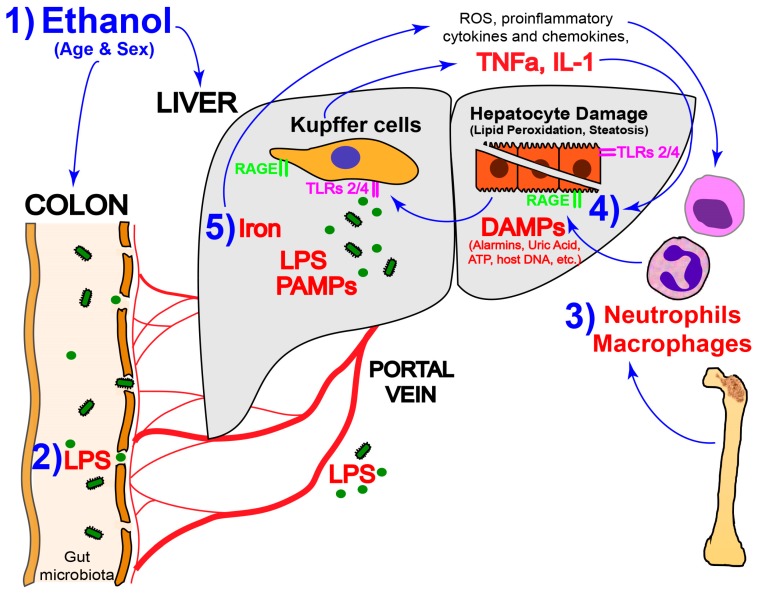
Key events contributing to the pathogenesis of alcoholic liver disease (ALD). (1) Aging and being a female enhance the vulnerability to ALD; (2) Lipopolysaccharide (LPS) translocation from the gut to the portal blood, which activates Kupffer cells (KC) to produce tumor necrosis factor α (TNFα), interleukin (IL)-1β and IL-18 via Toll-like receptors (TLRs) and other receptors still to be determined; (3) increased neutrophils and infiltration of bone marrow-derived macrophages that induce lipid peroxidation and hepatocyte steatosis, necrosis and apoptosis; (4) alcohol metabolism generates reduced nicotinamide adenine dinucleotide (NADH), which stimulates synthesis of excess fatty acids contributing to steatosis; and (5) iron deposition in the liver, which increases reactive oxygen species (ROS) production, leading to a proinflammatory microenvironment, therefore enhancing the severity of ALD. ATP: adenosine triphosphate; DAMPs: damage-associated molecular patterns; PAMPs: pathogen-associated molecular patterns.

The American Association for the Study of Liver Diseases and the European Association for the Study of Liver Diseases guidelines recommend corticosteroids as the first-line treatment in severe alcoholic hepatitis and pentoxifylline as the alternate in patients with ongoing infections or acute renal failure [109]. Still, they both recognize that current treatment options for patients with severe forms of AH and those who do not achieve abstinence, are suboptimal. Likewise, fine-tuning the inflammatory response in ALD and the lack of preclinical mouse models that recapitulate the inflammatory component of human ALD are current challenges in the field. Different mediators such as ROS, iron, neutrophils, inflammasome components, DAMPs and PAMPs have a central functional role in the pathogenesis of ALD (Figure 1). In parallel, relevant conditions such as gut leakiness [110], dysbiosis [111] and endotoxemia [112] factor into the pathogenesis of ALD and need to be thoroughly studied. Besides the progress in improving the quality of life in patients with ALD, there is still a pressing need to develop therapeutic interventions to abrogate the proinflammatory effect of antigens, bacterial-derived products and proinflammatory cytokines.

## References

[1] Roh Y.S., Zhang B., Loomba R., Seki E. (2015). TLR2 and TLR9 contribute to alcohol-mediated liver injury through induction of CXCL1 and neutrophil infiltration. Am. J. Physiol. Gastrointest. Liver Physiol..

[2] Joshi K., Kohli A., Manch R., Gish R. (2016). Alcoholic liver disease: High risk or low risk for developing hepatocellular carcinoma?. Clin. Liver Dis..

[3] Stickel F., Moreno C., Hampe J., Morgan M.Y. (2017). The genetics of alcohol dependence and alcohol-related liver disease. J. Hepatol..

[4] Buch S., Stickel F., Trepo E., Way M., Herrmann A., Nischalke H.D., Brosch M., Rosendahl J., Berg T., Ridinger M. (2015). A genome-wide association study confirms PNPLA3 and identifies TM6SF2 and MBOAT7 as risk loci for alcohol-related cirrhosis. Nat. Genet..

[5] Viollet B., Guigas B., Leclerc J., Hebrard S., Lantier L., Mounier R., Andreelli F., Foretz M. (2009). AMP-activated protein kinase in the regulation of hepatic energy metabolism: From physiology to therapeutic perspectives. Acta Physiol..

[6] Bala S., Csak T., Saha B., Zatsiorsky J., Kodys K., Catalano D., Satishchandran A., Szabo G. (2016). The pro-inflammatory effects of miR-155 promote liver fibrosis and alcohol-induced steatohepatitis. J. Hepatol..

[7] Zeng T., Zhang C.L., Song F.Y., Zhao X.L., Xie K.Q. (2012). Garlic oil alleviated ethanol-induced fat accumulation via modulation of SREBP-1, PPAR-α, and CYP2E1. Food Chem. Toxicol..

[8] Altamirano J., Bataller R. (2011). Alcoholic liver disease: Pathogenesis and new targets for therapy. Nat. Rev. Gastroenterol. Hepatol..

[9] Zhang M., Wang C., Wang C., Zhao H., Zhao C., Chen Y., Wang Y., McClain C., Feng W. (2015). Enhanced AMPK phosphorylation contributes to the beneficial effects of *Lactobacillus rhamnosus* GG supernatant on chronic-alcohol-induced fatty liver disease. J. Nutr. Biochem..

[10] Garcia-Villafranca J., Guillen A., Castro J. (2008). Ethanol consumption impairs regulation of fatty acid metabolism by decreasing the activity of AMP-activated protein kinase in rat liver. Biochimie.

[11] Kirpich I.A., Miller M.E., Cave M.C., Joshi-Barve S., McClain C.J. (2016). Alcoholic liver disease: Update on the role of dietary fat. Biomolecules.

[12] Teli M.R., Day C.P., Burt A.D., Bennett M.K., James O.F. (1995). Determinants of progression to cirrhosis or fibrosis in pure alcoholic fatty liver. Lancet.

[13] Stickel F. (2015). Alcoholic cirrhosis and hepatocellular carcinoma. Adv. Exp. Med. Biol..

[14] Sancho-Bru P., Altamirano J., Rodrigo-Torres D., Coll M., Millan C., Jose Lozano J., Miquel R., Arroyo V., Caballeria J., Gines P. (2012). Liver progenitor cell markers correlate with liver damage and predict short-term mortality in patients with alcoholic hepatitis. Hepatology.

[15] Dubuquoy L., Louvet A., Lassailly G., Truant S., Boleslawski E., Artru F., Maggiotto F., Gantier E., Buob D., Leteurtre E. (2015). Progenitor cell expansion and impaired hepatocyte regeneration in explanted livers from alcoholic hepatitis. Gut.

[16] Odena G., Chen J., Lozano J.J., Altamirano J., Rodrigo-Torres D., Affo S., Morales-Ibanez O., Matsushita H., Zou J., Dumitru R. (2016). LPS-TLR4 pathway mediates ductular cell expansion in alcoholic hepatitis. Sci. Rep..

[17] Laursen T.L., Stoy S., Deleuran B., Vilstrup H., Gronbaek H., Sandahl T.D. (2016). The damage-associated molecular pattern HMGB1 is elevated in human alcoholic hepatitis, but does not seem to be a primary driver of inflammation. APMIS.

[18] Lackner C., Spindelboeck W., Haybaeck J., Douschan P., Rainer F., Terracciano L., Haas J., Berghold A., Bataller R., Stauber R.E. (2016). Histological parameters and alcohol abstinence determine long-term prognosis in patients with alcoholic liver disease. J. Hepatol..

[19] Garcia-Saenz-de-Sicilia M., Duvoor C., Altamirano J., Chavez-Araujo R., Prado V., de Lourdes Candolo-Martinelli A., Holanda-Almeida P., Becerra-Martins-de-Oliveira B., Fernandez-de-Almeida S., Bataller R. (2016). A Day-4 Lille Model Predicts Response to Corticosteroids and Mortality in Severe Alcoholic Hepatitis. Am. J. Gastroenterol..

[20] Rehm J., Samokhvalov A.V., Shield K.D. (2013). Global burden of alcoholic liver diseases. J. Hepatol..

[21] Scaglione S., Kliethermes S., Cao G., Shoham D., Durazo R., Luke A., Volk M.L. (2015). The epidemiology of cirrhosis in the united states: A population-based study. J. Clin. Gastroenterol..

[22] Lemoinne S., Cadoret A., Rautou P.E., El Mourabit H., Ratziu V., Corpechot C., Rey C., Bosselut N., Barbu V., Wendum D. (2015). Portal myofibroblasts promote vascular remodeling underlying cirrhosis formation through the release of microparticles. Hepatology.

[23] Leung T.M., Nieto N. (2013). CYP2E1 and oxidant stress in alcoholic and non-alcoholic fatty liver disease. J. Hepatol..

[24] Bankoglu E.E., Tschopp O., Schmitt J., Burkard P., Jahn D., Geier A., Stopper H. (2016). Role of PTEN in Oxidative Stress and DNA Damage in the Liver of Whole-Body Pten Haplodeficient Mice. PLoS ONE.

[25] Zhou X., Han D., Xu R., Wu H., Qu C., Wang F., Wang X., Zhao Y. (2016). Glycine protects against high sucrose and high fat-induced non-alcoholic steatohepatitis in rats. Oncotarget.

[26] Park J.H., Lee D.H., Park M.S., Jung Y.S., Hong J.T. (2016). CCR5 deficiency exacerbates alcoholic fatty liver disease through pro-inflammatory cytokines and chemokines-induced hepatic inflammation. J. Gastroenterol. Hepatol..

[27] Lee S.J., Lee D.E., Kang J.H., Nam M.J., Park J.W., Kang B.S., Lee D.S., Lee H.S., Kwon O.S. (2016). New potential biomarker proteins for alcoholic liver disease identified by a comparative proteomics approach. J. Cell. Biochem..

[28] Li H.H., Tyburski J.B., Wang Y.W., Strawn S., Moon B.H., Kallakury B.V., Gonzalez F.J., Fornace A.J. (2014). Modulation of fatty acid and bile acid metabolism by peroxisome proliferator-activated receptor α protects against alcoholic liver disease. Alcohol. Clin. Exp. Res..

[29] Kwon H.J., Won Y.S., Park O., Chang B., Duryee M.J., Thiele G.E., Matsumoto A., Singh S., Abdelmegeed M.A., Song B.J. (2014). Aldehyde dehydrogenase 2 deficiency ameliorates alcoholic fatty liver but worsens liver inflammation and fibrosis in mice. Hepatology.

[30] Holmuhamedov E.L., Czerny C., Beeson C.C., Lemasters J.J. (2012). Ethanol suppresses ureagenesis in rat hepatocytes: Role of acetaldehyde. J. Biol. Chem..

[31] Mani V., Arivalagan S., Siddique A.I., Namasivayam N. (2016). Antioxidant and anti-inflammatory role of zingerone in ethanol-induced hepatotoxicity. Mol. Cell. Biochem..

[32] Comporti M., Signorini C., Leoncini S., Gardi C., Ciccoli L., Giardini A., Vecchio D., Arezzini B. (2010). Ethanol-induced oxidative stress: Basic knowledge. Genes Nutr..

[33] Portari G.V., Ovidio P.P., Deminice R., Jordao A.A. (2016). Protective effect of treatment with thiamine or benfotiamine on liver oxidative damage in rat model of acute ethanol intoxication. Life Sci..

[34] Bradford B.U., Rusyn I. (2005). Swift increase in alcohol metabolism (SIAM): Understanding the phenomenon of hypermetabolism in liver. Alcohol.

[35] Zhu H., Jia Z., Misra H., Li Y.R. (2012). Oxidative stress and redox signaling mechanisms of alcoholic liver disease: Updated experimental and clinical evidence. J. Dig. Dis..

[36] Lieber C.S. (2005). Metabolism of alcohol. Clin. Liver Dis..

[37] Baraona E., Lieber C.S. (1979). Effects of ethanol on lipid metabolism. J. Lipid Res..

[38] Howarth D.L., Vacaru A.M., Tsedensodnom O., Mormone E., Nieto N., Costantini L.M., Snapp E.L., Sadler K.C. (2012). Alcohol disrupts endoplasmic reticulum function and protein secretion in hepatocytes. Alcohol. Clin. Exp. Res..

[39] Cederbaum A.I. (2012). Alcohol metabolism. Clin. Liver Dis..

[40] Baraona E., Abittan C.S., Dohmen K., Moretti M., Pozzato G., Chayes Z.W., Schaefer C., Lieber C.S. (2001). Gender differences in pharmacokinetics of alcohol. Alcohol. Clin. Exp. Res..

[41] Mansouri A., Fromenty B., Berson A., Robin M.A., Grimbert S., Beaugrand M., Erlinger S., Pessayre D. (1997). Multiple hepatic mitochondrial DNA deletions suggest premature oxidative aging in alcoholic patients. J. Hepatol..

[42] Fromenty B., Grimbert S., Mansouri A., Beaugrand M., Erlinger S., Rotig A., Pessayre D. (1995). Hepatic mitochondrial DNA deletion in alcoholics: Association with microvesicular steatosis. Gastroenterology.

[43] Ramirez T., Li Y.M., Yin S., Xu M.J., Feng D., Zhou Z., Zang M., Mukhopadhyay P., Varga Z.V., Pacher P. (2016). Aging aggravates alcoholic liver injury and fibrosis in mice by downregulating sirtuin 1 expression. J. Hepatol..

[44] Kim I.H., Kisseleva T., Brenner D.A. (2015). Aging and liver disease. Curr. Opin. Gastroenterol..

[45] Tajiri K., Shimizu Y. (2013). Liver physiology and liver diseases in the elderly. World J. Gastroenterol..

[46] Ma T.Y., Hollander D., Dadufalza V., Krugliak P. (1992). Effect of aging and caloric restriction on intestinal permeability. Exp. Gerontol..

[47] Farhadi A., Banan A., Fields J., Keshavarzian A. (2003). Intestinal barrier: An interface between health and disease. J. Gastroenterol. Hepatol..

[48] Datz C., Felder T.K., Niederseer D., Aigner E. (2013). Iron homeostasis in the metabolic syndrome. Eur. J. Clin. Investig..

[49] Winterbourn C.C. (1995). Toxicity of iron and hydrogen peroxide: The fenton reaction. Toxicol. Lett..

[50] Houglum K., Filip M., Witztum J.L., Chojkier M. (1990). Malondialdehyde and 4-hydroxynonenal protein adducts in plasma and liver of rats with iron overload. J. Clin. Investig..

[51] Pietrangelo A. (2016). Mechanisms of iron hepatotoxicity. J. Hepatol..

[52] Varghese J., James J.V., Sagi S., Chakraborty S., Sukumaran A., Ramakrishna B., Jacob M. (2016). Decreased hepatic iron in response to alcohol may contribute to alcohol-induced suppression of hepcidin. Br. J. Nutr..

[53] Pietrangelo A. (2016). Iron and the liver. Liver Int..

[54] Ambade A., Satishchandran A., Gyongyosi B., Lowe P., Szabo G. (2016). Adult mouse model of early hepatocellular carcinoma promoted by alcoholic liver disease. World J. Gastroenterol..

[55] Li M., He Y., Zhou Z., Ramirez T., Gao Y., Gao Y., Ross R.A., Cao H., Cai Y., Xu M. (2016). MicroRNA-223 ameliorates alcoholic liver injury by inhibiting the IL-6-p47^phox^-oxidative stress pathway in neutrophils. Gut.

[56] Bukong T.N., Iracheta-Vellve A., Saha B., Ambade A., Satishchandran A., Gyongyosi B., Lowe P., Catalano D., Kodys K., Szabo G. (2016). Inhibition of spleen tyrosine kinase activation ameliorates inflammation, cell death, and steatosis in alcoholic liver disease. Hepatology.

[57] Stoy S., Dige A., Sandahl T.D., Laursen T.L., Buus C., Hokland M., Vilstrup H. (2015). Cytotoxic t lymphocytes and natural killer cells display impaired cytotoxic functions and reduced activation in patients with alcoholic hepatitis. Am. J. Physiol. Gastrointest. Liver Physiol..

[58] Stadlbauer V., Mookerjee R.P., Wright G.A., Davies N.A., Jurgens G., Hallstrom S., Jalan R. (2009). Role of Toll-like receptors 2, 4, and 9 in mediating neutrophil dysfunction in alcoholic hepatitis. Am. J. Physiol. Gastrointest. Liver Physiol..

[59] Andrews K., Abdelsamed H., Yi A.K., Miller M.A., Fitzpatrick E.A. (2013). TLR2 regulates neutrophil recruitment and cytokine production with minor contributions from TLR9 during hypersensitivity pneumonitis. PLoS ONE.

[60] Petrasek J., Iracheta-Vellve A., Csak T., Satishchandran A., Kodys K., Kurt-Jones E.A., Fitzgerald K.A., Szabo G. (2013). Sting-IRF3 pathway links endoplasmic reticulum stress with hepatocyte apoptosis in early alcoholic liver disease. Proc. Natl. Acad. Sci. USA.

[61] Krysko D.V., Kaczmarek A., Krysko O., Heyndrickx L., Woznicki J., Bogaert P., Cauwels A., Takahashi N., Magez S., Bachert C. (2011). TLR-2 and TLR-9 are sensors of apoptosis in a mouse model of doxorubicin-induced acute inflammation. Cell Death Differ..

[62] Huebener P., Pradere J.P., Hernandez C., Gwak G.Y., Caviglia J.M., Mu X., Loike J.D., Jenkins R.E., Antoine D.J., Schwabe R.F. (2015). The HMGB1/rage axis triggers neutrophil-mediated injury amplification following necrosis. J. Clin. Investig..

[63] Bangert A., Andrassy M., Muller A.M., Bockstahler M., Fischer A., Volz C.H., Leib C., Goser S., Korkmaz-Icoz S., Zittrich S. (2016). Critical role of RAGE and HMGB1 in inflammatory heart disease. Proc. Natl. Acad. Sci. USA.

[64] Zhou H., Yu M., Zhao J., Martin B.N., Roychowdhury S., McMullen M.R., Wang E., Fox P.L., Yamasaki S., Nagy L.E. (2016). IRAKM-Mincle axis links cell death to inflammation: Pathophysiological implications for chronic alcoholic liver disease. Hepatology.

[65] Hirsiger S., Simmen H.P., Werner C.M., Wanner G.A., Rittirsch D. (2012). Danger signals activating the immune response after trauma. Mediat. Inflamm..

[66] Gross S.R., Sin C.G., Barraclough R., Rudland P.S. (2014). Joining S100 proteins and migration: For better or for worse, in sickness and in health. Cell Mol. Life Sci..

[67] Bertheloot D., Latz E. (2017). HMGB1, IL-1α, IL-33 and S100 proteins: Dual-function alarmins. Cell. Mol. Immunol..

[68] Rider P., Carmi Y., Guttman O., Braiman A., Cohen I., Voronov E., White M.R., Dinarello C.A., Apte R.N. (2011). IL-1α and IL-1β recruit different myeloid cells and promote different stages of sterile inflammation. J. Immunol..

[69] Dinarello C.A., Simon A., van der Meer J.W. (2012). Treating inflammation by blocking interleukin-1 in a broad spectrum of diseases. Nat. Rev. Drug Discov..

[70] Lodder J., Denaes T., Chobert M.N., Wan J., El-Benna J., Pawlotsky J.M., Lotersztajn S., Teixeira-Clerc F. (2015). Macrophage autophagy protects against liver fibrosis in mice. Autophagy.

[71] Varga T., Mounier R., Horvath A., Cuvellier S., Dumont F., Poliska S., Ardjoune H., Juban G., Nagy L., Chazaud B. (2016). Highly dynamic transcriptional signature of distinct macrophage subsets during sterile inflammation, resolution, and tissue repair. J. Immunol..

[72] Pradere J.P., Kluwe J., De Minicis S., Jiao J.J., Gwak G.Y., Dapito D.H., Jang M.K., Guenther N.D., Mederacke I., Friedman R. (2013). Hepatic macrophages but not dendritic cells contribute to liver fibrosis by promoting the survival of activated hepatic stellate cells in mice. Hepatology.

[73] Gao B., Bataller R. (2011). Alcoholic liver disease: Pathogenesis and new therapeutic targets. Gastroenterology.

[74] Arriazu E., Ge X., Leung T.M., Magdaleno F., Lopategi A., Lu Y., Kitamura N., Urtasun R., Theise N., Antoine D.J. (2016). Signalling via the osteopontin and high mobility group box-1 axis drives the fibrogenic response to liver injury. Gut.

[75] Ge X., Leung T.M., Arriazu E., Lu Y., Urtasun R., Christensen B., Fiel M.I., Mochida S., Sørensen E.S., Nieto N. (2014). Osteopontin binding to lipopolysaccharide lowers tumor necrosis factor-α and prevents early alcohol-induced liver injury in mice. Hepatology.

[76] Ge X., Lu Y., Leung T.M., Sorensen E.S., Nieto N. (2013). Milk osteopontin, a nutritional approach to prevent alcohol-induced liver injury. Am. J. Physiol. Gastrointest. Liver Physiol..

[77] Kolaczkowska E., Kubes P. (2013). Neutrophil recruitment and function in health and inflammation. Nat. Rev. Immunol..

[78] Amanzada A., Moriconi F., Mansuroglu T., Cameron S., Ramadori G., Malik I.A. (2014). Induction of chemokines and cytokines before neutrophils and macrophage recruitment in different regions of rat liver after TAA administration. Lab. Investig..

[79] De Filippo K., Henderson R.B., Laschinger M., Hogg N. (2008). Neutrophil chemokines KC and macrophage-inflammatory protein-2 are newly synthesized by tissue macrophages using distinct TLR signaling pathways. J. Immunol..

[80] Fraczek J., Kim T.W., Xiao H., Yao J., Wen Q., Li Y., Casanova J.L., Pryjma J., Li X. (2008). The Kinase Activity of IL-1 Receptor-associated Kinase 4 is Required for Interleukin-1 Receptor/Toll-like Receptor-induced TAK1-dependent NFκB Activation. J. Biol. Chem..

[81] Kim T.W., Staschke K., Bulek K., Yao J., Peters K., Oh K.H., Vandenburg Y., Xiao H., Qian W., Hamilton T. (2007). A critical role for IRAK4 kinase activity in Toll-like receptor-mediated innate immunity. J. Exp. Med..

[82] Yao J., Kim T.W., Qin J., Jiang Z., Qian Y., Xiao H., Lu Y., Qian W., Gulen M.F., Sizemore N. (2007). Interleukin-1 (IL-1)-induced TAK1-dependent versus MEKK3-dependent NFκB activation pathways bifurcate at IL-1 receptor-associated kinase modification. J. Biol. Chem..

[83] Franchi L., Eigenbrod T., Munoz-Planillo R., Nunez G. (2009). The inflammasome: A caspase-1-activation platform that regulates immune responses and disease pathogenesis. Nat. Immunol..

[84] Szabo G., Petrasek J. (2015). Inflammasome activation and function in liver disease. Nat. Rev. Gastroenterol. Hepatol..

[85] Martinon F., Burns K., Tschopp J. (2002). The Inflammasome: A Molecular Platform Triggering Activation of Inflammatory Caspases and Processing of proIL-β. Mol. Cell.

[86] Schroder K., Tschopp J. (2010). The inflammasomes. Cell.

[87] Hoque R., Farooq A., Ghani A., Gorelick F., Mehal W.Z. (2014). Lactate reduces liver and pancreatic injury in Toll-like receptor- and inflammasome-mediated inflammation via GPR81-mediated suppression of innate immunity. Gastroenterology.

[88] Prochnicki T., Mangan M.S., Latz E. (2016). Recent insights into the molecular mechanisms of the NLRP3 inflammasome activation. F1000Research.

[89] Cui K., Yan G., Xu C., Chen Y., Wang J., Zhou R., Bai L., Lian Z., Wei H., Sun R. (2015). Invariant NKT cells promote alcohol-induced steatohepatitis through interleukin-1β in mice. J. Hepatol..

[90] Dinarello C.A. (1998). Interleukin-1β, interleukin-18, and the interleukin-1β converting enzyme. Ann. N. Y. Acad. Sci..

[91] Mehta R., Neupane A., Wang L., Goodman Z., Baranova A., Younossi Z.M. (2014). Expression of NALPs in adipose and the fibrotic progression of non-alcoholic fatty liver disease in obese subjects. BMC Gastroenterol..

[92] Bracey N.A., Gershkovich B., Chun J., Vilaysane A., Meijndert H.C., Wright J.R., Fedak P.W., Beck P.L., Muruve D.A., Duff H.J. (2014). Mitochondrial NLRP3 protein induces reactive oxygen species to promote Smad protein signaling and fibrosis independent from the inflammasome. J. Biol. Chem..

[93] Lozano-Ruiz B., Bachiller V., Garcia-Martinez I., Zapater P., Gomez-Hurtado I., Moratalla A., Gimenez P., Bellot P., Frances R., Such J. (2015). Absent in melanoma 2 triggers a heightened inflammasome response in ascitic fluid macrophages of patients with cirrhosis. J. Hepatol..

[94] Richardson M.B., Williams S.J. (2014). Mcl and mincle: C-type lectin receptors that sense damaged self and pathogen-associated molecular patterns. Front. Immunol..

[95] Wree A., Marra F. (2016). The inflammasome in liver disease. J. Hepatol..

[96] Iracheta-Vellve A., Petrasek J., Satishchandran A., Gyongyosi B., Saha B., Kodys K., Fitzgerald K.A., Kurt-Jones E.A., Szabo G. (2015). Inhibition of sterile danger signals, uric acid and ATP, prevents inflammasome activation and protects from alcoholic steatohepatitis in mice. J. Hepatol..

[97] Ioannou G.N., Van Rooyen D.M., Savard C., Haigh W.G., Yeh M.M., Teoh N.C., Farrell G.C. (2015). Cholesterol-lowering drugs cause dissolution of cholesterol crystals and disperse Kupffer cell crown-like structures during resolution of NASH. J. Lipid Res..

[98] Hendrikx T., Bieghs V., Walenbergh S.M., van Gorp P.J., Verheyen F., Jeurissen M.L., Steinbusch M.M., Vaes N., Binder C.J., Koek G.H. (2013). Macrophage specific caspase-1/11 deficiency protects against cholesterol crystallization and hepatic inflammation in hyperlipidemic mice. PLoS ONE.

[99] Lundback P., Lea J.D., Sowinska A., Ottosson L., Furst C.M., Steen J., Aulin C., Clarke J.I., Kipar A., Klevenvall L. (2016). A novel high mobility group box 1 neutralizing chimeric antibody attenuates drug-induced liver injury and postinjury inflammation in mice. Hepatology.

[100] Antoine D.J., Jenkins R.E., Dear J.W., Williams D.P., McGill M.R., Sharpe M.R., Craig D.G., Simpson K.J., Jaeschke H., Park B.K. (2012). Molecular forms of HMGB1 and keratin-18 as mechanistic biomarkers for mode of cell death and prognosis during clinical acetaminophen hepatotoxicity. J. Hepatol..

[101] Antoine D.J., Williams D.P., Kipar A., Laverty H., Park B.K. (2010). Diet restriction inhibits apoptosis and HMGB1 oxidation and promotes inflammatory cell recruitment during acetaminophen hepatotoxicity. Mol. Med..

[102] Kang R., Zhang Q., Hou W., Yan Z., Chen R., Bonaroti J., Bansal P., Billiar T.R., Tsung A., Wang Q. (2014). Intracellular HMGB1 inhibits inflammatory nucleosome release and limits acute pancreatitis in mice. Gastroenterology.

[103] Chen R., Fu S., Fan X.G., Lotze M.T., Zeh H.J., Tang D., Kang R. (2015). Nuclear DAMP Complex-mediatedRAGE-Dependent Macrophage Cell Death. Biochem. Biophys. Res. Commun..

[104] Chen R., Hou W., Zhang Q., Kang R., Fan X.G., Tang D. (2013). Emerging role of high-mobility group box 1 (HMGB1) in liver diseases. Mol. Med..

[105] Wu C.X., Sun H., Liu Q., Guo H., Gong J.P. (2012). LPS induces HMGB1 relocation and release by activating the NF-κB-CBP signal transduction pathway in the murine macrophage-like cell line RAW264.7. J. Surg. Res..

[106] Kim Y.M., Park E.J., Kim J.H., Park S.W., Kim H.J., Chang K.C. (2016). Ethyl pyruvate inhibits the acetylation and release of HMGB1 via effects on SIRT1/STAT signaling in LPS-activated RAW264.7 cells and peritoneal macrophages. Int. Immunopharmacol..

[107] Ge X., Antoine D.J., Lu Y., Arriazu E., Leung T.M., Klepper A.L., Branch A.D., Fiel M.I., Nieto N. (2014). High mobility group box-1 (HMGB1) participates in the pathogenesis of alcoholic liver disease (ALD). J. Biol. Chem..

[108] Farooq A., Hoque R., Ouyang X., Farooq A., Ghani A., Ahsan K., Guerra M., Mehal W.Z. (2014). Activation of *N*-methyl-d-aspartate receptor downregulates inflammasome activity and liver inflammation via a β-arrestin-2 pathway. Am. J. Physiol. Gastrointest. Liver Physiol..

[109] O’Shea R.S., Dasarathy S., McCullough A.J., Practice Guideline Committee of the American Association for the Study of Liver Diseases, Practice Parameters Committee of the American College of Gastroenterology (2010). Alcoholic liver disease. Hepatology.

[110] Mir H., Meena A.S., Chaudhry K.K., Shukla P.K., Gangwar R., Manda B., Padala M.K., Shen L., Turner J.R., Dietrich P. (2016). Occludin deficiency promotes ethanol-induced disruption of colonic epithelial junctions, gut barrier dysfunction and liver damage in mice. Biochim. Biophys. Acta.

[111] Ferrere G., Wrzosek L., Cailleux F., Turpin W., Puchois V., Spatz M., Ciocan D., Rainteau D., Humbert L., Hugot C. (2016). Fecal microbiota manipulation prevents dysbiosis and alcohol-induced liver injury in mice. J. Hepatol..

[112] Rao R. (2009). Endotoxemia and gut barrier dysfunction in alcoholic liver disease. Hepatology.

